# Training medical students in motivational interviewing using a blended learning approach: a proof-of-concept study

**DOI:** 10.3389/fpsyg.2023.1204810

**Published:** 2023-07-20

**Authors:** Rebecca Erschens, Bettina Fahse, Teresa Festl-Wietek, Anne Herrmann-Werner, Katharina E. Keifenheim, Stephan Zipfel, Andreas J. Fallgatter, Kerstin Velten-Schurian

**Affiliations:** ^1^University Medical Hospital Tuebingen, Internal Medicine, Department of Psychosomatic Medicine and Psychotherapy, Tübingen, Germany; ^2^Tübingen Institute for Medical Education (TIME), Faculty of Medicine, Tübingen, Germany; ^3^German Center for Mental Health (DZPG), Tuebingen, Germany; ^4^Tübingen Center for Mental Health, Department of Psychiatry and Psychotherapy, University of Tübingen, Tübingen, Germany

**Keywords:** motivational interviewing, blended learning, medical training, student training, medical curriculum

## Abstract

**Background:**

Difficulties in implementing behavior change in patients with chronic diseases are common in clinical practice. Motivational interviewing (MI) helps clinicians to support patients in overcoming ambivalence while maintaining self-determination. The inclusion of MI in German medical training curricula is still rare. Furthermore, the effects of systematic teaching of MI, especially via blended learning, have hardly been investigated.

**Methods:**

Medical students participated in three curricular events related to MI, consisting of instructional videos and theoretical and practical components in a blended learning format. The aim of the study was to investigate the effect of teaching MI in students’ medical education. A controlled, non-randomized study was conducted with an intervention group and a control group. Both groups completed questionnaires on their experience and knowledge related to MI, completed a knowledge test and rated their satisfaction with the course. MI was taught in the 6th semester of medical coursework as part of a psychosomatic course, in the 8th semester during a psychiatry course and in the 9th semester during a weekly psychiatry clerkship.

**Results:**

Data from the intervention group (*n* = 35) and control group (*n* = 14) were analyzed, with 65.7% of students participating in all three parts of the curriculum. Overall interest in learning MI was high, with *M* = 2.92 (*SD* = 1.00). The results indicate a greater increase in knowledge over time in the intervention group. The majority (62.86%) stated that the curriculum was relevant to their future career. Free-form text responses indicated a high level of satisfaction with practical relevance.

**Conclusion:**

This study demonstrates the usefulness of an MI curriculum for medical students. The integration of MI into medical curricula is a promising curricular addition to improve doctor-patient communication. Future research should address patient perceptions of MI competencies and the persistence of acquired competencies.

## Introduction

### Ambivalence and changing behavior in chronic disease

As current prevalence rates show, around 40% of the population in Germany over the age of 16 can be considered chronically ill (Bundesministerium Für Gesundheit et al., 2021). This is reflected in high rates of hypertension, diabetes, chronic obstructive pulmonary disease, cardiovascular disease, chronic back pain and obesity (Rathmann et al., 2018; Hoebel et al., 2019; Güthlin et al., 2020). In addition to these physical diseases, prevalence rates also include mental disorders, the most common of which are anxiety, affective disorders and addictive disorders (Jacobi et al., 2014). The impact of chronic diseases is associated with a high burden of disease, reduced quality of life for patients and high health care costs (Statistisches Bundesamt, 2017).

Triggers and perpetuators of chronic disease include unhealthy lifestyles and poor health choices, which often lead to a complex treatment process and complicate doctor-patient interactions (Frost et al., 2018). Unhealthy behaviors are often difficult to change or abandon due to long-standing habits and short-term positive effects. Individuals therefore experience strong ambivalence caused by the simultaneous occurrence of conflicting desires, thoughts or feelings for or against behavior change. Behavior change is crucial in the recovery process of various mental and physical illnesses, as a healthy lifestyle is associated with a significantly reduced risk of chronic disease (Ford et al., 2009). Thus, it is possible to prevent or positively influence the development of a chronic disease by changing behavior toward a healthier lifestyle, e.g., through more exercise and a healthier diet (Hu and Willett, 2002; Vainio et al., 2002) or abstaining from nicotine (Mannino and Buist, 2007). Furthermore, internal and external motivation can be considered key variables for behavior change in patients (Ryan and Deci, 2017).

Healthcare workers and physicians should therefore be involved in improving patients’ motivation to change, as professional support can facilitate active participation in managing their own health and making decisions that promote self-care (Barbosa et al., 2021). But how can physicians support their patients in changing a certain behavior—especially when, due to its positive short-term consequences and its habitual character, the behavior in question is hard to give up? The ambivalence which arises from these conditions often prevent patients from making a decision.

### The concept of motivational interviewing

Addressing this issue, Miller and Rollnick (1991), Miller and Rollnick (2004), and Miller and Rollnick (2012) developed motivational interviewing (MI). This approach provides well-defined negotiation techniques as well as a specific attitude, called the motivational interviewing spirit, that help build a reliable physician-patient relationship as well as evoke and enhance patients’ intrinsic motivation for behavior change (e.g., utilization of an indicated medical treatment). Notably, MI aims to foster patients’ intrinsic (vs. extrinsic) motivation. Therefore, performing MI does not imply convincing the patient to do what the physician thinks is best. Especially in the presence of ambivalence, confronting patients or persuading them toward a change in behavior tends to provoke arguments against that very change. Such a confrontation highlights the advantages of the status quo and the disadvantages of the behavior change, making actual behavior change less probable.

In contrast, practicing MI means supporting the patient in identifying behavior changes that are desirable from their perspective as well as in finding out which motives or aspects they perceive to be helpful in pursuing the specific change. According to Miller and Rollnick (2012), the interviewer should take into account the following ethical values: nonmaleficence, beneficence, autonomy and justice. These values provide the ethical framework for MI. This framework implies, for example, that the interviewer does not support the patient in pursuing harmful behaviors, but that the interviewer accepts if the patient decides against a healthy behavior change. Thus, MI can augment the traditional role of a physician as an expert—someone who informs the patient of the diagnosis and recommends treatment options—and can open up a communication style that focuses on the patient’s perspective, particularly when a patient shows ambivalence regarding a certain behavior change.

### Effectiveness of motivational interviewing

MI was initially developed for the treatment of addictive disorders. Since then, the field of application has gradually been extended to other mental and physical diseases. In a Cochrane Review (Smedslund et al., 2011), MI was shown to significantly reduce the amount of alcohol consumption. In comparison to other interventions investigated in that study, MI was equally or more effective. Positive effects of MI have also been shown in the treatment of eating disorders (Treasure et al., 1999; Hettema et al., 2005; Cassin et al., 2008; Geller and Dunn, 2011; Westra et al., 2011) as well as overweight and obesity (Van Dorsten, 2007; Armstrong et al., 2011; Teufel et al., 2011).

In a meta-analysis (Lundahl et al., 2013), MI was found to be associated with significant effects regarding behavior change in several health-related behavior categories. Among others, MI enhanced the participation of pain patients in workshops imparting pain management strategies (Habib et al., 2005), reduced risk behavior in adolescents who previously had been treated due to an injury (Johnston et al., 2002), increased health-promoting behaviors in adolescents with HIV (Naar-King et al., 2008), improved the attitude of patients with type 2 diabetes toward health-promoting behavior changes (Rubak et al., 2009) and reduced regeneration of caries mediated by mothers’ preventive behaviors (Weinstein et al., 2006).

These findings demonstrate the usefulness of MI for widespread use in the health sector and the associated need for knowledge transfer about MI.

### Communication skills and motivational interviewing in medical education

Training in basic communication skills is already an integral part of medical training at various faculties (Smith et al., 2007). In addition, the use of simulated patients for in-depth practical training is widely accepted and perceived to be of great value for students (Kaplonyi et al., 2017), as is the use of peer role play as a simulation-based training method (Gelis et al., 2020). Both the German Master Plan 2020 for medical education and the national catalog of competency-based learning objectives for medicine reinforce the importance of doctor-patient communication skills, including MI (Wissing, 2018).

Research has demonstrated the success of teaching MI techniques in the medical field, both in the training of medical students and in the training of fully qualified doctors (Frost et al., 2018; Kaltman and Tankersley, 2020). A review by Kaltman and Tankersley (2020) found that participation in MI courses led to increased knowledge and improved practical skills in medical students. A recent study by Jacobs et al. (2021) showed that MI training in pre-medical students has a positive effect on knowledge and specific skills. Another review by Dunhill et al. (2014) showed that training in MI is particularly effective when it is “intensive,” i.e., when it involves several sessions or is integrated into a longitudinal curriculum, and when interactive exercises are an integral part of the training.

Several studies were able to show that MI can be successfully taught through training and workshops (e.g., Evangeli et al., 2009; Britt and Blampied, 2010; Walters et al., 2010) for an overview see Schwalbe et al. (2014) and that training MI improves knowledge and practical skills (Madson et al., 2009). In terms of didactic modality, MI is best learned in communication workshops with feedback and targeted coaching (Miller et al., 2004) while focusing on the underlying assumptions and *spirit* of MI rather than on specific techniques (Miller and Moyers, 2006). Embodying the *spirit* is associated with an increased likelihood of internalizing other relevant MI values and behaviors such as acceptance, respect for autonomy, empathy and warmth (Moyers et al., 2005). The reported prevalence and high use of MI in the health care system highlights the need for MI to be taught at an early stage of medical education.

### Motivational interviewing and the blended learning approach

Blended learning describes the combination of face-to-face teaching with online materials and courses (Delialioglu and Yildirim, 2007) and has been shown to have better outcomes in terms of knowledge acquisition compared to traditional learning and online-only learning in health education (Morton et al., 2016; Westerlaken et al., 2019; Vallée et al., 2020).

The benefits of both formats, such as the efficiency and flexibility of online materials and the interaction with peers and tutors afforded by in-person learning, can be combined in one approach. Meta-analytic results suggest that the use of blended learning in medical education and training is significantly superior to traditional teaching in terms of growth in theoretical knowledge (Vallée et al., 2020) and satisfaction with the curriculum (Li et al., 2019).

### Objective and research questions

In line with the need for more high-quality research on MI in education (Frost et al., 2018), our study integrates the factors identified in the literature as helpful and practical, such as workshops with feedback and targeted coaching, spirit in the early stages of medical education. We complement the literature by implementing our curriculum in a blended learning format, both to optimize time management and to ensure that it can be used independently of the face-to-face seminar.

To the authors’ knowledge, the only published German study on MI training for medical students in a blended learning format is the pilot study of the present study (Keifenheim et al., 2019). In the pilot study, significant improvements in subjective and objective knowledge as well as (subjective) practical skills were achieved after the first of three parts of the MI curriculum. The present study investigates the success of MI training in a blended learning format (i.e., a combination of lecture, simulation patient videos, face-to-face practical sessions and role-play scenarios) for medical students in their 6th to 9th semester onwards as part of their mandatory medical courses.

The research questions were:

Q1: Does participation in an MI curriculum offered in a blended learning format lead to an increase in students’ subjectively rated theoretical knowledge of MI?

Q2: Does participation in an MI curriculum offered in a blended learning format lead to an increase in students’ subjectively assessed practical skills in MI?

Q3: Does participation in an MI curriculum offered in a blended learning format lead to an increase in students’ objectively assessed theoretical knowledge of MI?

## Materials and methods

### Study design

This evaluation was conducted at a German medical university faculty among medical students between the 6th and 9th semesters, prior to the COVID-19 pandemic. Using a longitudinal pre-post design, students were compared with assignment to the intervention or control group (non-randomized) and the two time points of measurement (T0/T2) as independent variables. Only data from subjects who met the following criteria were used for analysis: (i) completed questionnaires at T0 (6th-semester medical students) and T2 (9th-semester medical students) and (ii) participated in at least two of the three videos and two of the three practical components.

### The MI curriculum

The MI curriculum consisted of three parts (MI 1–3). While the first part focused on MI rationale, MI spirit and the physician-patient relationship, the second and third parts concentrated on specific MI techniques. MI spirit was briefly summarized at the beginning of MI 2 and MI 3 and constituted the basis of all role-play interactions when training specific MI techniques. In order to show and train students on different uses, each part of the curriculum highlighted a specific field of application (eating disorders, addictive disorders and health-promoting behavior).

The materials were prepared by a psychotherapist and resident psychiatrist who has acquired theoretical and practical knowledge through literature studies and several MI workshops, as well as several years of experience in the application of MI in addictive disorders, health-promoting behaviors and eating disorders. MI trainers were psychotherapists and physicians with at least 1 year of professional experience. They studied MI literature, curriculum videos and instructions for the practical sessions. In addition, they received a practical training of about 3 h with the psychotherapist or physician. Throughout the course of the curriculum, trainers were supervised by the psychotherapist or physician. Table 1 summarizes the content of the curriculum.

**Table 1:** Content overview of the MI curriculum for each semester.

	Semester		
	6th semester (MI part 1)	8th semester (MI part 2)	9th semester (MI part 3)
Teaching course	seminar “Psychosomatic Medicine and Psychotherapy”	seminar “Psychiatry”	weekly internship “Psychiatry”
Different aspects of MI	History and development of MI	Review MI 1	Review MI 2
	MI Rationale (e.g., change talk, sustain talk)	Evoking change talk (e.g., importance ruler)	Evoking change talk (e.g., elicit-provide-elicit)
	MI spirit (OARS)^1^	Enhancing change talk	Responding to sustain talk and discord
	Building up physician-patient relationship	Evoking hope and confidence	Limitations
	Focusing and developing goals	Developing a change plan	
Focused health issue	Eating disorders	Addictive disorders	Health promoting behavior

1 OARS: Open questions, affirmations, reflective listening, and summary reflections.

### Participants and study procedure

The link to the online questionnaire was sent to 6th-semester medical students of the respective medical faculty (*control group* in the winter semester, *intervention group* in the summer semester) (time T0). The intervention group then took part in a compulsory first course of the newly introduced curriculum on MI (MI 1).

The course (3 units, 135 min) on MI was integrated into an existing seminar (“Psychosomatic Medicine and Psychotherapy”) as one of six sessions. In advance, the students were asked to watch online teaching and demonstration videos (2 units, 90 min). In the so-called “teaching videos,” theoretical knowledge was conveyed to the students in the form of a recorded lecture. For the “therapy demonstration videos,” example conversations (with simulation patients) were filmed in which a doctor applies MI. In the 8th and 9th semesters, the students in the intervention group took part in further seminars on MI (MI 2 and MI 3). Here, too, a 1.5-h session on MI was integrated into an existing course (in the 8th-semester seminar “Psychiatry” and in the 9th-semester weekly internship “Psychiatry”). In addition, the students were asked to watch teaching and demonstration videos on an online platform (approximately 30 min).

**Figure 1:**
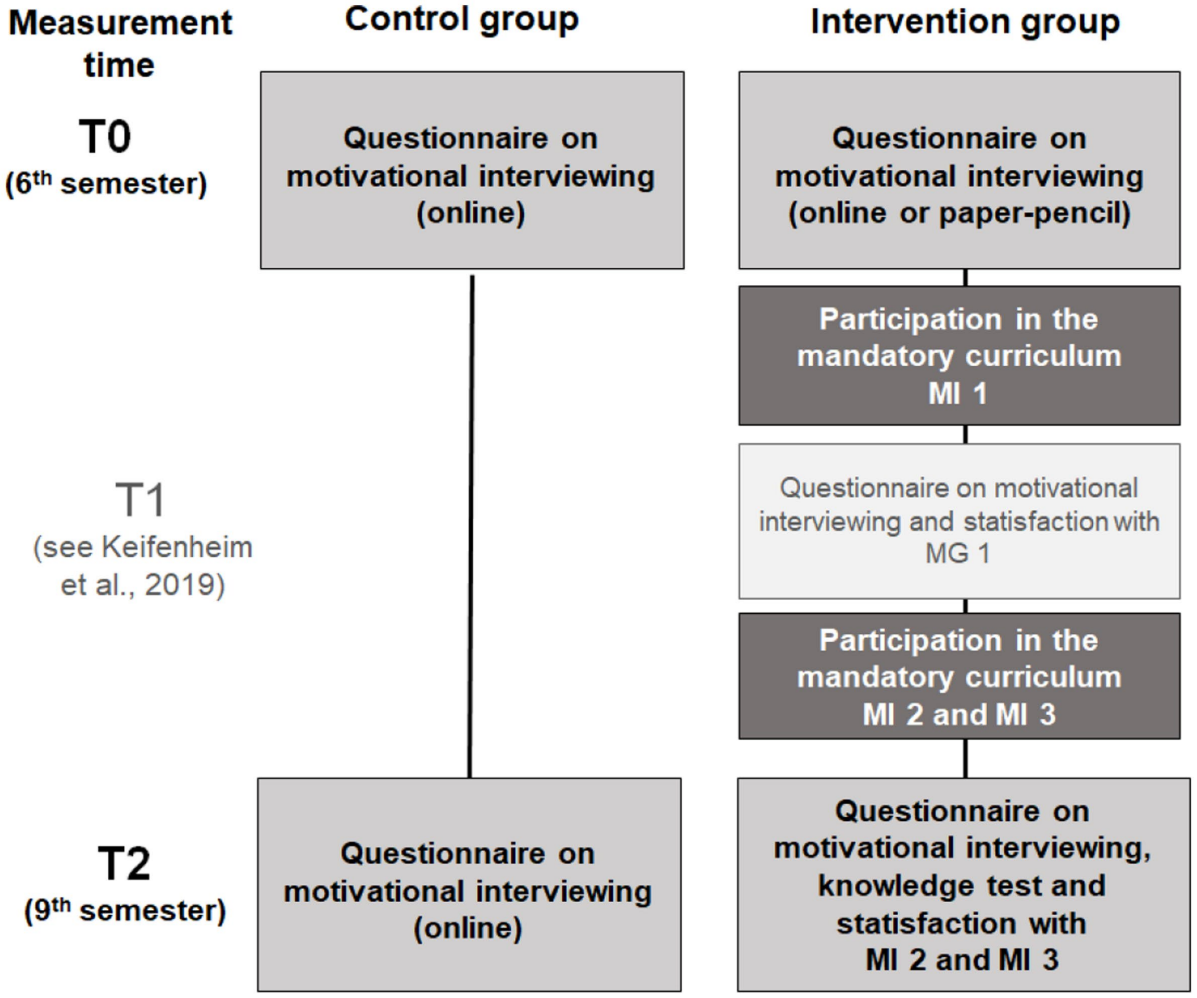
Visualization of the procedure of the MI curriculum and timing of the respective questionnaires in control (left) and intervention group (right). T1 represents the preceding pilot study of this publication by Keifenheim et al. (2019) incorporating a needs assessment as well as the evaluation of the first part of the curriculum at time T2.

The control group studied in the 6th semester according to the original timetable, without the opportunity to participate in the MI sessions. At the end of the 9th semester, all participants in the control group who had completed the questionnaire at time T0 received an email with a link to a very similar, slightly adapted questionnaire (time T2). Figure 1 summarizes the study procedure in detail.

### Evaluation instruments

The Questionnaire on Motivational Interviewing (see Figure 1) consisted of a “knowledge test” section, an “assessment of subjective theoretical knowledge and practical skills” section and a section on “satisfaction with the curriculum.”

#### Knowledge test

The knowledge test consisted of 12 questions on MI methods adapted from Poirier et al. (2004) in a multiple-choice format with five possible answers (one of which was “correct”). The questions related to the topics covered in the curriculum (see Table 1). Four multiple-choice questions were asked for each curriculum unit MI 1–3. Table 2 shows three examples for the respective curriculum units.

**Table 2:** Illustration of examples from the knowledge test questionnaire for MI1 - MI3.

MI Unit	Which statement is accurate?	Multiple choice answers^1^
6th semester (MI part 1)	In motivational interviewing, doctors should...	a) ... argue for a change in behavior.
		b) emphasize the need to accept the diagnosis (e.g., “You have an eating disorder”).
		c) …Give priority to expert knowledge.
		d) **…adapt their intervention to the patient’s readiness to change**
		e) …offer solutions.
8th semester (MI part 2)	In motivational interviewing, if a doctor doubts that the intentions expressed by the patient (e.g., starting alcohol-specific treatment) are serious, ...	a) .. he/she should gently confront the patient.
		b) **.. he/she should appreciate the intention to change and ask for the reasons for this statement**
		c) .. he/she should show appreciation, but make it clear that a lack of motivation could significantly limit the success of the therapy.
		d) .. he/she should not react to this and try to establish a “real” motivation in the further conversation.
		e) he/she should reconsider the therapy goals with the patient.
9th semester (MI part 3)	By exploring the barriers to behavior change ..	a) .. the patient’s unrealistic expectations of success should be relativized.
		b) **.. motivation for change can be enhanced. ..**
		c) …the motivation to change is usually reduced.
		d) .. the chances of actually changing behavior should be assessed.
		e) .. the doctor-patient relationship in particular should be strengthened.

1 In bold: correct answer.

#### Rating on subjective theoretical knowledge and practical skills

In addition to demographic items (age, gender, semester, specialization) and items on previous training in communication skills in general and MI in particular, this section included a subjective assessment of knowledge and skills in MI and an objective test of theoretical knowledge. At T0, the questionnaire also included an item on interest in MI. Subjective ratings of theoretical knowledge, interest and practical skills in MI were made using Likert-scale items ranging from 0 (non-existing) to 4 (very high). Theoretical knowledge and interest were each assessed with one item, and practical skills were assessed with a total score and four subscales for individual methods. Again, a scale from 0 (non-existent) to 4 (very high) was used.

The subscales related to the specific competencies, as well as their corresponding questions, were:

- *Practice of the therapeutic stance of MI* (“How do you currently assess your practical skills in applying the specific therapeutic stance of motivational interviewing?”)
- *Basic interview skills based on Miller and Rollnick* (“How do you currently rate your practical skills in using the basic interview skills of ‘open questions’, ‘confirmation’, ‘simple and complex reflection’ and ‘summarizing’?”)
- *Eliciting change talk* (“How do you currently rate your practical skills in initiating and reinforcing change talk with patients?”)
- *Rolling with resistance* (“How do you currently rate your practical skills in rolling with patient resistance, e.g., to indicated treatment?”).

#### Curriculum satisfaction

In addition, participants in the intervention group completed a curriculum satisfaction questionnaire at T2. The curriculum was rated on a scale from 1 to 6 at three levels:

- Overall
- In terms of its relevance to the future medical profession
- With regard to the three specific sub-components of “instructional videos,” “therapy videos” and “practical exercises” (in each case with regard to their comprehensibility and perceived relevance for learning MI)

In addition, the use of the e-learning format was assessed. Participants also had the opportunity to give positive and negative feedback in a free-form text response.

### Data analysis

SPSS for Windows (version 22.0) was used for quantitative analysis. Data were analyzed visually and by Kolmogorov–Smirnov test under the assumption of normal distribution. However, if a non-normal distribution was suspected, non-parametric Mann–Whitney U test was also used. Homogeneity of variance was tested using Levene’s test. Descriptive statistics of participants between the two groups were tested for differences in means and distributions using *t*-tests and *chi-squared* tests. Analyses of variance were calculated using T0 and T2 as within-subject factors and group (control vs. intervention) as a between-subject factor to examine the impact of the MI curriculum. The Mann–Whitney U test was used to test for homogeneity of variance. Partial eta squared (η^2^) was used as the effect size for ANOVA and Pearson’s correlation coefficient *r* for *t-*tests. Correlations were calculated using Pearson’s correlation coefficient *r*, Spearman’s correlation coefficient *r*_s_ or Kendall’s correlation coefficient τ depending on the scale level. At the nominal scale level, possible correlation was tested using Fisher’s exact chi-squared test and the non-parametric Mann–Whitney U test. *p*-values below 0.05 were considered significant.

For qualitative analysis, feedback in the free-response format was categorized according to qualitative content analysis (Mayring and Fenzl, 2010). Missing answers in the multiple-choice knowledge test were defined as “wrong,” similar to the procedure in a written exam. In the case of missing questionnaire data, the item, but not the participant, was excluded from the analysis of variance. If the questionnaire was canceled, the participant was excluded from the data analysis. Students who attended fewer than two parts of the seminar or watched fewer than two information videos were also excluded.

## Results

### Sample characteristics

#### Participation and dropout

**Figure 2:**
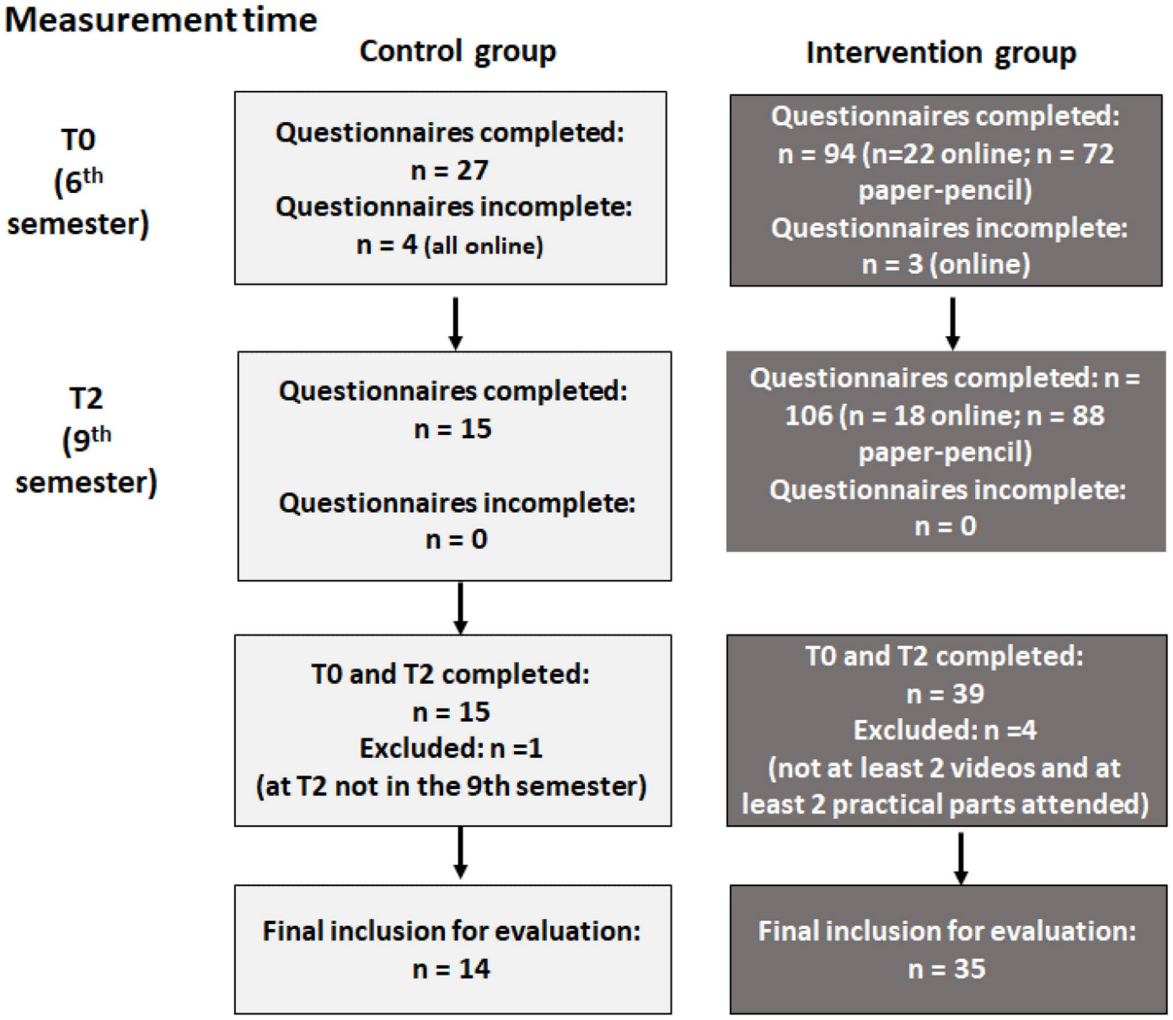
Representation of participant statistics at each measurement point in the control (left) and intervention (right) group.

At baseline (T0), the medical students were in their 6th semester. Participation in the study was conditional on consent within the intervention group (IG) and participation in the MI curriculum. Of the 91 students in the IG who completed the questionnaire at baseline, 39 also completed the questionnaires at T2. Of these, 35 met the evaluation criteria (participated in at least two practice days and viewed at least two videos) and could therefore be included in the evaluation of the study. Of the 23 students in the control group (CG) who completed the questionnaire at T0, 15 students also completed the questionnaire at T2. One person in the CG—presumably due to a sabbatical/research semester—was still in the 8th semester at T2, and thus in the IG cohort, and was therefore excluded from the analysis. Consequently, 14 subjects of the CG were included in the evaluation of the study. Figure 2 provides an overview of the progression of the participants.

#### Demographics

The IG consisted of 21 women and 14 men aged between 22 and 31 years (*M* = 24.51, *SD* = 2.76). The 14 subjects of the CG included 10 women and 4 men aged between 23 and 34 years (*M* = 27.29, *SD* = 3.69). A homogeneity test showed no significant difference in gender distribution between the groups. Participants in the two groups differed significantly in terms of age. A detailed overview can be found in Table 3. We interpret the difference to mean that the CG started their studies in a summer semester, which, as the German A-Level takes place in the spring/summer, tends to result in older students. Furthermore, the variable age had no influence on the assessment of subjective theoretical and practical knowledge or on the objective theoretical knowledge test (all *r* < |0.18|, all *p* > 0.281). See Table 3 for demographic details.

**Table 3:** Characteristics of the total sample at T0: demographics, number of semesters attended and general interest and prior experience with MI.

Characteristics	Control group (CG)	Intervention group (IG)	*p*
Age, *M (SD)*	27.3 (3.69)	24.5 (2.76)	0.006
Sex (female)	*n* = 10	*n* = 21	0.541
Semester	6th: *n* = 13 7th: *n* = 1	7th: n = 35	*Not applicable*
Ever heard of MI	*n* = 11: no *n* = 3: yes	*n* = 30: no *n* = 5: yes	0.541
Interest in MI, *M* (*SD*)	2.57 (0.85)	3.06 (1.03)	0.124

#### Association between practice intensity and assessment of subjective theoretical knowledge and objective knowledge

Of the 35 participants in the IG, 23 (65.7%) participated in all three practice components, 11 (31.4%) participated in MI 1 and MI 2 and one (2.9%) participated in MI 1 and MI 3. Of the 35 IG participants, 20 (57.1%) watched all of the videos from the practice components, 11 (31.4%) only watched the videos from MI 1 and MI 2, two (5.7%) watched the videos from MI 1 and MI 3 and two (5.7%) watched the videos from MI 2 and MI 3. The number of practical days completed (two or three) correlated neither with the increase in knowledge in objective theoretical knowledge (*r* = 0.18, *p* = 0.150) nor with subjective theoretical knowledge (*r* = 0.23, *p* = 0.177). In contrast, the number of completed practical days correlated significantly with the increase in practical knowledge (*r* = 0.37, *p* = 0.039). After applying the Bonferroni correction, this association was no longer significant. The correlation between the number of videos watched (two or three) and the increase in objective theoretical knowledge was also significant (*r* = 0.28, *p* = 0.050), but not after Bonferroni correction. The number of videos watched (two or three) did not correlate significantly with the increase in subjective theoretical knowledge (*r* = 0.13, *p* = 0.459) or practical knowledge (*r* = 0.30, *p* = 0.106).

### Interest and previous experience with MI

In the total sample, interest in learning MI was, on average, high to very high (*M* = 2.92; *SD* = 1.00; 0 [none] to 4 [very high]). Prior to the start of the study, 83.7% of the subjects had participated in one or two general communication training sessions offered as part of their medical training. With regard to MI in particular, 98% of participants reported that they had received no training or input in this area prior to the start of their studies. In line with this, students rated their theoretical knowledge (*M* = 0.24; *SD* = 0.48; 0 [none] to 4 [very high]) and practical skills in MI (*M* = 0.37; *SD* = 0.64; 0 [none] to 4 [very high]) as rather low.

### Knowledge growth

#### Subjective theoretical knowledge growth

**Figure 3:**
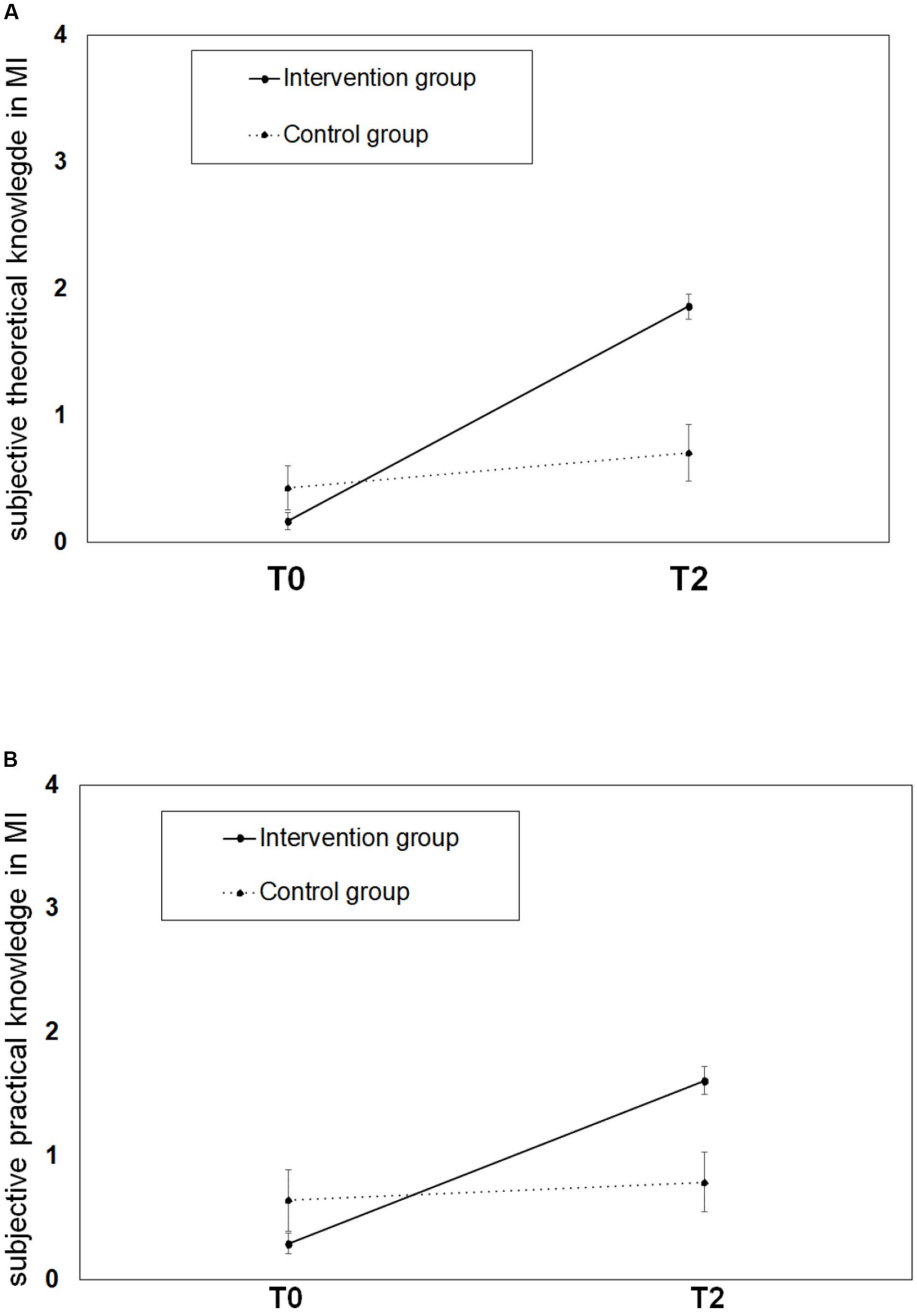
Illustration of student’s subjective theoretical and practical knowledge growth on MI in the IG and the CG before and after participation in the MI-curriculum. **(A)** Shows the growth in *subjective theoretical knowledge* assessed by questionnaire within IG and CG. Measurement points T0 and T2 are plotted on the x-axis. **(B)** Shows the growth in *subjective practical* knowledge within IG and CG. Measurement points T0 and T2 are plotted on the x-axis.

A two-factor analysis of variance (group x time) with repeated measures revealed a main effect of measurement time (*F*(1,47) = 126.85, *p* < 0.001, η^2^ = 0.73). Both groups showed significantly more subjective theoretical knowledge at T2 (*M* = 1.53, *SD* = 0.84) than at T0 (*M* = 0.24, *SD* = 0.48). Prior to attending the MI curriculum (T0), students in the intervention group (IG) reported significantly lower subjective theoretical knowledge than students in the control group (CG), with *M* = 0.17, *SD* = 0.38, T2: *M* = 1.86, *SD* = 0.60. Due to variance heterogeneity, an additional Mann–Whitney U test was performed. This test showed no significant difference between the two groups at T0 (IG: *Mdn* = 0, CG: *Mdn* = 0, *U* = 196.50, *z* = −1.48, *p* = 0.186). The interaction between group and time was significant (*F*(1,47) = 63.97, *p* < 0.001, η^2^ = 0.58). Students in the IG experienced a significantly greater enhancement in subjective theoretical knowledge than students in the CG (see Figure 3A).

#### Subjective practical knowledge growth in general

A two-factor analysis of variance (group x time) with repeated measures revealed a main effect of measurement time (*F*(1,43) = 54.01, *p* < 0.001, η*^2^* = 0.56). Both groups showed significantly more subjective practical knowledge at T2 (*M* = 1.36, *SD* = 0.80) than at T0 (*M* = 0.40, *SD* = 0.65). The main effect of group assignment was not significant (*F*(1,43) = 1.50, *p* = 0.227, η*^2^* = 0.03). The expected interaction between group and time point reached significance (*F*(1,43) = 35.01, *p* < 0.001, η*^2^* = 0.45). Students in the IG reported a significantly greater increase in subjective practical knowledge than students in the CG. Due to the lack of homogeneity of variance and normal distribution, an additional Mann–Whitney U test was performed and showed no difference between the two groups at T0 (IG: *Mdn* = 0, CG: *Mdn* = 0, *U* = 178.00, *z* = −1.16, *p* = 0.246), but a significant difference at T2 (IG: *Mdn* = 2, CG: *Mdn* = 1, *U* = 93.50, *z* = −3.25, *p* < 0.001; see Figure 3B).

#### Analysis of specific therapeutic competencies

Here, only the interaction effects are reported.

- *Practice of the therapeutic stance of MI*. There was an interaction effect (*F*(1,46) = 29.05, *p* < 0.001, η^2^ = 0.39) whereby participants in the IG (T0: *M* = 0.59, *SD* = 0.78, T2: *M* = 2.00, *SD* = 0.55) showed a greater increase in subjective knowledge of basic therapeutic attitudes than participants in the CG (T0: *M* = 0.86, *SD* = 1.10, T2: *M* = 0.71, *SD* = 0.83).
- *Basic interview skills based on Miller and Rollnick*. An interaction effect was found (*F*(1,46) = 18.13, *p* < 0.001, η^2^ = 0.28) indicating that the IG (T0: *M* = 1.74, *SD* = 0.90, T2: *M* = 2.38, *SD* = 0.65) reported a greater subjective improvement in their MI-specific interviewing skills than the CG (T0: *M* = 2.14, *SD* = 0.95, T2: *M* = 1.43, *SD* = 1.16).
- *Eliciting change talk*. Following an interaction effect (*F*(1,46) = 20.43, *p* < 0.001, η^2^ = 0.31), participants in the IG (T0: *M* = 0.76, *SD* = 0.70, T2: *M* = 1.79, *SD* = 0.54) reported a greater enhancement in subjective skills related to eliciting change talk than participants in the CG (T0: *M* = 1.50, *SD* = 0.86, T2: *M* = 1.36, *SD* = 0.75).
- *Rolling with resistance*. There was an interaction effect (*F*(1,46) = 8.48, *p* = 0.006, η^2^ = 0.16) whereby participants in the IG (T0: *M* = 1.12, *SD* = 0.69, T2: *M* = 1.71, *SD* = 0.63) reported a greater increase in subjective ability to deal with resistance than the CG (T0: *M* = 1.50, *SD* = 0.65, T2: *M* = 1.21, *SD* = 0.89).

#### Objective theoretical knowledge growth

**Figure 4:**
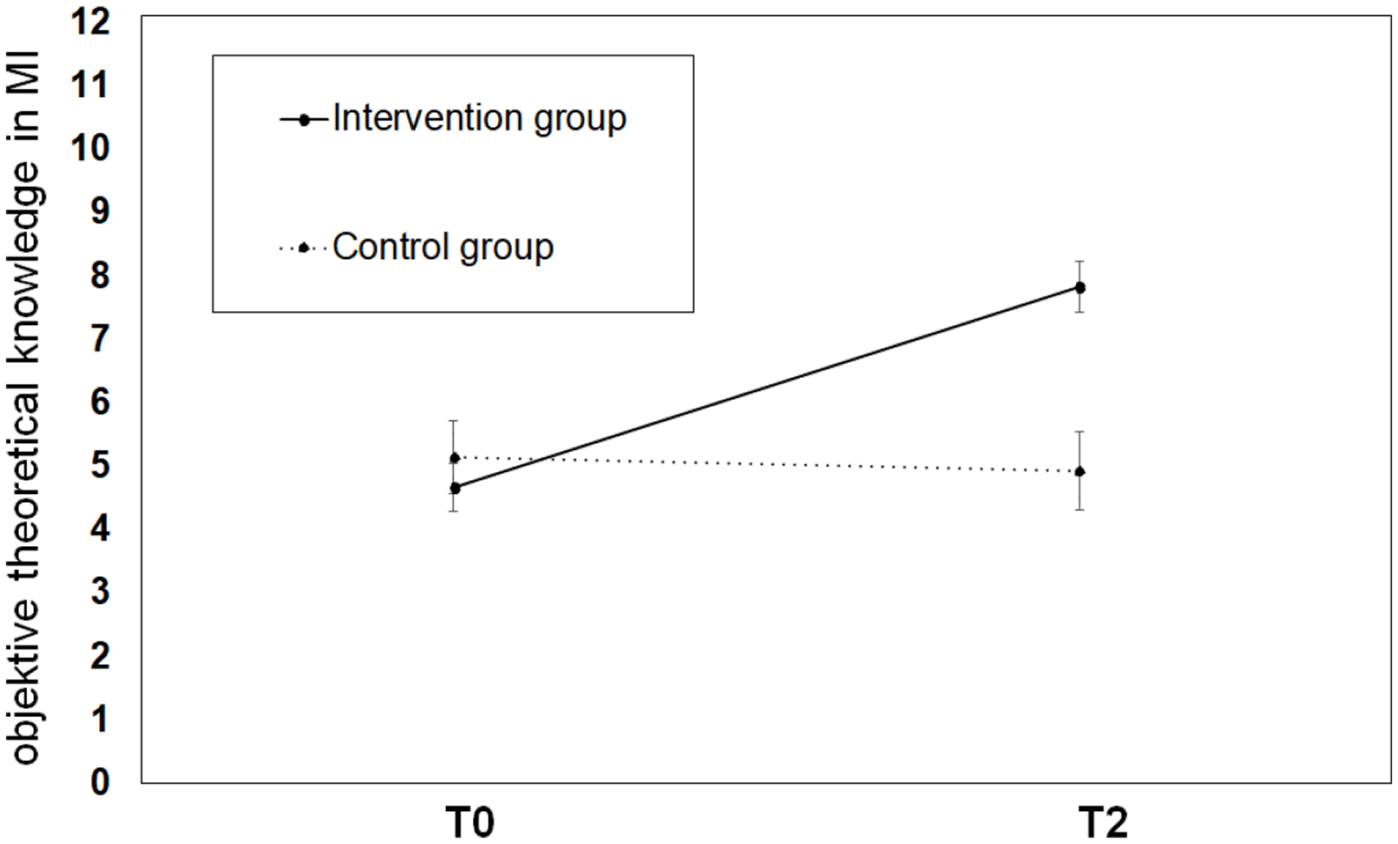
Illustration of student’s objective knowledge growth on MI in the IG and the CG before and after participation in the MI-curriculum. Different levels of objective theoretical knowledge growth assessed by a knowledge test within IG and CG are plotted on the y-axis, measurement points T0 and T2 are plotted on the x-axis.

There was a main effect of time (*F*(1,47) = 15.15, *p* < 0.001, η^2^ = 0.24) showing that the score on the MI knowledge test increased significantly in both groups from time T0 (*M* = 4.80, *SD* = 2.24) to time T2 (*M* = 7.00, *SD* = 2.68). There was a trend toward a main effect of the groups (*F*(1,47) = 3.87, *p* = 0.055, η^2^ = 0.08). The expected interaction effect (*F*(1,47) = 19.86, *p* < 0.001, η^2^ = 0.30) showed that participants in the IG (T0: *M* = 4.66, *SD* = 2.24, T2: *M* = 7.83, *SD* = 2.36) achieved a greater increase in objective knowledge than participants in the CG (T0: *M* = 5.14, *SD* = 2.14, T2: *M* = 4.93, *SD* = 2.34). Figure 4 provides a graphical illustration.

### Curriculum evaluation

#### Total

The 35 students in the intervention group rated the curriculum with a mean score of *M* = 2.80, *SD* = 0.90.

#### Importance for later professional activity

Most students (62.86%) agreed or strongly agreed that the content of the curriculum was very relevant to their later professional work as doctors. Evaluated in school grades, the relevance of the lecture videos received a mean rating of 3.12 (*SD* = 1.25), the relevance of the therapy videos a mean rating of 2.80 (*SD* = 1.39) and the relevance of the practical exercises a mean rating of 2.41 (*SD* = 1.19).

#### Follow-up of the curriculum

The majority of students (65.72%) thought that the curriculum should be maintained.

#### Comprehensibility of teaching materials

Evaluated in school grades, the lecture videos received a mean rating of 1.89 (*SD* = 0.93), the therapy videos a mean rating of 1.74 (*SD* = 0.78) and the practical exercises a mean rating of 2.00 (*SD* = 1.02). There were no significant differences between the mean scores of the three teaching formats regarding comprehensibility (*F*(2,62) = 2.03, *p* = 0.139, η^2^ = 0.06).

#### Qualitative feedback

In order to gain a deeper understanding of the students’ needs and desires for the MI curricula, their free-text responses were qualitatively evaluated.

They reported the following benefits of the curricula:

- The topic in general/the theoretical background/learning conversation techniques
- The practical relevance and the exercises with the simulated patients
- The information from the videos
- The practical orientation

Students suggested the following changes to the curricula:

- There should be more practical exercises with (simulation) patients and more feedback on the exercises.
- The MI curriculum should be taught in one block/semester and should be more integrated with other courses.
- The videos should be replaced or supplemented by a live performance by teachers with patients in the lessons.

## Discussion

To the best of our knowledge, this is the first study to examine the implementation of a blended format motivational interviewing (MI) curriculum in a German human medicine curriculum. The results of the variance analysis show that participation in the curriculum was successful in terms of MI. Students who participated in the curriculum improved their subjective and objective knowledge of MI. Improvements were also shown in the subcategories regarding the MI-specific techniques of “basic therapeutic attitude,” “basic interview skills,” “eliciting change talk,” and “rolling with resistance” (Miller and Rollnick, 2012). Despite the rather small sample size, the effects were robust with high effect sizes. The curriculum used was shown to be effective in increasing students’ subjective skills and objective knowledge compared to a control group.

After completing the entire curriculum, students rated the curriculum with an average grade of 2.8. The results showed that after the first component MI 1 after the 6th semester (Keifenheim et al., 2019), the students seemed to be slightly more satisfied with the curriculum than after completing all three components of the curriculum. Medical students rated the curriculum with a grade of 2.2 regarding satisfaction after the first MI, and one of the reasons for this could be the videos: Compared to the survey in T1 (*cf.* Keifenheim et al., 2019), the videos lost popularity in the quantitative evaluation in T2. Both lecture videos were rated “good” (2.26) at T2 in terms of their relevance for learning MI and “satisfactory” (3.12) after T2. The therapy videos also dropped one point in relevance, from 1.83 to 2.80. In comparison, the practical exercises declined less.

The qualitative results (free-form text responses) showed that the videos were rated well by many students, but ambivalently to negatively by others. Students felt that the patient videos lacked authenticity and that the lecture videos should be replaced by a “real” lecture. Students in subsequent semesters (6th vs. 8th and 9th semesters) assessed the relevance of MI at least equally important for their later professional career. Possibly, authentic patient contact, where students experienced MI as helpful, may have resulted in this assessment (see Nortvig et al., 2018). However, it should be noted that the videos in MI 1 were watched at home, whereas in MI 2 and MI 3 they were watched together with the lecturers in the face-to-face course to ensure that the students were familiar with the content. This lack of “voluntariness” and the implementation of the curriculum in the compulsory course could possibly explain the decrease in satisfaction toward the three modules.

Another explanation could be that the anticipated advantages of the videos, such as the possibility of repeated viewing or increased time for practical exercises (see Phillips et al., 2016), were not considered by the students to be as important as the advantages of a live lecture. Ultimately, this means that an in-person lecture and sufficient time for practice are desirable, but this would require more staff and also more teaching time.

The participants’ wish for more authentic videos of example conversations might reflect, that this group of people is not yet accustomed to learn from schematic and complexity reduced interaction sequences. Possibly, this, for teaching reasons, intended gap between the video interactions and reality, reduced the credibility of the effectiveness of MI (“Can it even work in real situations?”) in the students’ perception. This, in turn, might have downgraded the evaluation of the videos and the extent to which at least some of the students were able to learn from them. Still, in our opinion, complexity reduced examples are, as in numerous other fields of teaching, common and helpful for a step by step training of complex abilities. Considering the feedback of the participants in our study we would suggest, to make this purpose of schematic videos more transparent for the students. In addition, students should be asked for more specific feedback concerning the videos, so that more specific improvements can be derived.

In summary, the results indicate that there are different groups with different needs in terms of the specific design of the MI curriculum. For the overall group of medical students, it may be more appropriate to offer an introduction as a compulsory seminar with reduced content and time. For students with greater interest and commitment, further seminars could be offered on a voluntary basis to facilitate the deepening of knowledge and practical skills in MI.

Several authors have discussed how MI teaching can best be implemented. Fuhrmann et al. (2022) investigated interactive learning content where students could choose between different responses or answers. This led to a natural development of patient-doctor communication and could be a useful option for implementation in the MI curriculum. Despite all the benefits described, developing an online format is time consuming and often an organizational challenge (Phillips et al., 2016). For future synergy, it is thus essential that higher education institutions network in order to exchange experiences and share learning content. As suggested by Hurlocker et al. (2020), a methodologically sound instrument is necessary to assess MI competencies. The use of the same assessment system in all higher education institutions would allow valid comparisons.

In the present study, initial interest and satisfaction did not influence knowledge growth. Neither knowledge growth nor interest had a significant effect on satisfaction. It is possible that learning outcomes and satisfaction are more dependent on individual values or goals, as these are also reflected in later subject choices (Bexelius et al., 2016). Further research should address individual differences in order to better tailor the curriculum to students and achieve even higher learning outcomes and satisfaction.

This study provides evidence that could be applied to the durability and transferability of learned skills into clinical practice. To date, there is limited and mixed evidence in the current literature on MI (Kaltman and Tankersley, 2020). According to a meta-analytic finding, MI skills tend to decline approximately 6 months after training (Schwalbe et al., 2014); however, Miller and Rollnick (2004) have shown robust skill gains up to 12 months after training. This has implications for whether booster sessions should be used and when the use of boosters might be most useful (Fu et al., 2015; Schechter et al., 2021). Due to the therapeutic impact of MI on common chronic diseases in modern society that place a heavy burden on the healthcare system, introducing medical students to this topic is proving to be highly relevant (Rubak et al., 2009; Armstrong et al., 2011; Lundahl et al., 2013).

As a final note, the term “resistance” was critically discussed and deemed misleading in the third edition of Motivational Interviewing – Helping People Change (Miller and Rollnick, 2012). When designing the curriculum, we discussed this issue thoroughly and decided to mention the term “resistance” because the respective interactions are often perceived in that way, especially by non-experienced clinicians. In order to explain and evaluate the phenomenon of (perceived) resistance in line with what Miller and Rollnick (2012) outline, we added the expression “dealing with difficult situations,” and we pointed out that patient behaviors that cause a feeling of resistance in the interviewer can have different reasons. For instance, the change in question may seem unreachable for the patient, the positive consequences of change may seem too far away or too insecure, the patient may feel general hopelessness as a consequence of many unsuccessful attempts and, last but not least, the interviewer may exhibit inadequate behavior.

### Limitations

There were also some limitations to this study. One limitation relates to the measurement tools used. As the questionnaires were not validated, but developed in-house, psychometric data on validity has limited interpretability. In addition, no objective assessment of practical skills was made. The authors are aware of the limited sample size in the control group, and future studies should pay attention to the selection of appropriate measurement instruments and assessment forms in general and also for raters such as the MITI or the MI-SCOPE (Hannöver et al., 2012; Kitzmann et al., 2019).

## Conclusion and further directions

This study provides evidence that teaching practical and theoretical knowledge of MI via a three-part curriculum in a blended learning format as part of mandatory medical courses can be successful. However, the results show that despite their learning success, students were only moderately satisfied with the curriculum. Therefore, there is still potential for future improvement, especially regarding the video formats, possibly through future inter-university exchanges. Further, the results of this study suggest that in the obligatory mode, a shorter and more basic training might be more appropriate while more in-depth training should be offered to students with a particular interest in MI.

## Data availability statement

The raw data supporting the conclusions of this article will be made available by the authors upon reasonable request, subject to data protection requirements.

## Ethics statement

The study was approved by the local ethics committee (No. 038/2016BO2). All participants gave informed consent to participate. As an incentive, six book vouchers worth €20 each were raffled among all participants at each measurement.

## Author contributions

RE: data analysis, data interpretation, revision of figures and tables, and writing the first draft and all revisions of the manuscript. BF: data acquisition, data analysis, interpretation of data, creating figures, and tables, substantial input to the first draft, the revisions of the manuscript and prepares a medical thesis in German language based on the presented data. TF-W: data interpretation and critical revision of manuscript. AH-W: study design, data interpretation, and critical revision of manuscript. KEK: study design, data acquisition, interpretation of data, and critical revision of manuscript. AJF: coordination and critical revision of manuscript. SZ: study design, coordination, and critical revision of manuscript. KV-S: study design, data acquisition, interpretation of data, and critical revision of manuscript. All authors contributed to study design, analysis and writing, and approved the final manuscript.

## Funding

This work was supported by the Medical Faculty Tübingen (PROFILplus program), Deutsche Forschungsgemeinschaft.

## Conflict of interest

The authors declare that the research was conducted in the absence of any commercial or financial relationships that could be construed as a potential conflict of interest.

## Publisher’s note

All claims expressed in this article are solely those of the authors and do not necessarily represent those of their affiliated organizations, or those of the publisher, the editors and the reviewers. Any product that may be evaluated in this article, or claim that may be made by its manufacturer, is not guaranteed or endorsed by the publisher.
